# *Aiptasia* sp. larvae as a model to reveal mechanisms of symbiont selection in cnidarians

**DOI:** 10.1038/srep32366

**Published:** 2016-09-01

**Authors:** Iliona Wolfowicz, Sebastian Baumgarten, Philipp A. Voss, Elizabeth A. Hambleton, Christian R. Voolstra, Masayuki Hatta, Annika Guse

**Affiliations:** 1 Centre for Organismal Studies (COS), Heidelberg University, Heidelberg 69120, Germany; 2 Graduate Program in Areas of Basic and Applied Biology (GABBA), University of Porto, Porto 4200-465, Portugal; 3 Red Sea Research Center, Division of Biological and Environmental Science and Engineering, King Abdullah University of Science and Technology (KAUST), Thuwal 23955-6900, Saudi Arabia; 4 Graduate School of Humanities and Sciences, Ochanomizu University, Tokyo 112-8610, Japan

## Abstract

Symbiosis, defined as the persistent association between two distinct species, is an evolutionary and ecologically critical phenomenon facilitating survival of both partners in diverse habitats. The biodiversity of coral reef ecosystems depends on a functional symbiosis with photosynthetic dinoflagellates of the highly diverse genus *Symbiodinium*, which reside in coral host cells and continuously support their nutrition. The mechanisms underlying symbiont selection to establish a stable endosymbiosis in non-symbiotic juvenile corals are unclear. Here we show for the first time that symbiont selection patterns for larvae of two *Acropora* coral species and the model anemone *Aiptasia* are similar under controlled conditions. We find that *Aiptasia* larvae distinguish between compatible and incompatible symbionts during uptake into the gastric cavity and phagocytosis. Using RNA-Seq, we identify a set of candidate genes potentially involved in symbiosis establishment. Together, our data complement existing molecular resources to mechanistically dissect symbiont phagocytosis in cnidarians under controlled conditions, thereby strengthening the role of *Aiptasia* larvae as a powerful model for cnidarian endosymbiosis establishment.

Coral reefs, the most biodiverse marine ecosystem on earth, depend on a functional symbiosis between corals and dinoflagellate algae to survive in nutrient poor waters. Intracellular dinoflagellates from the genus *Symbiodinium* transfer photosynthetic products to the coral host, thereby greatly contributing to corals’ nutrition in tropical habitats^1^. The genus *Symbiodinium* is very diverse^2^ and it has been known for decades that symbiont/host combinations are not random: corals establish symbiosis with some *Symbiodinium* types but not others, termed “symbiosis specificity”^3^,^4^. In the face of climate change, this phenomenon has attracted increasing attention because certain symbiont types may allow their host to cope with changing environments better than others^5^,^6^,^7^,^8^,^9^. Despite this ecological importance, the fundamental mechanisms underlying “symbiosis specificity” remain poorly understood.

Symbiont selectivity may be in part governed during early life stages: many coral species produce symbiont-free offspring that acquire symbionts from the environment^10^,^11^, and coral larvae often appear to favor homologous symbionts (i.e. those found in parents) over heterologous types^5^,^12^,^13^. Similar to other endosymbiotic relationships, establishment of coral symbiosis is thought to follow a “winnowing process”^14^,^15^ comprising a series of steps, each of which is crucial for a stable, specific symbiotic interaction. In coral larvae and juvenile polyps, the following steps are involved in symbiosis establishment: symbiont entrance into the gastric cavity, symbiont phagocytosis by endodermal host cells, symbiont integration into the host cells, initiation of nutrient transfer, and symbiont proliferation throughout the endodermal tissue of the host. Because adult corals may not be capable of continuously acquiring new symbionts from the environment, the symbiont population integrated during early life stages is most likely important for a functional interplay between symbionts and host cells^4^,^16^.

The molecular mechanisms underlying the steps in symbiont selection in juvenile corals are poorly understood. Likewise, how long-term hosting of foreign organisms affects host and symbiont cell physiologies, including gene expression, nutrient transfer, cell organization, and cell division, is largely unclear. Progress in understanding these processes mechanistically has been, and still is, slow because molecular tools for corals and their larvae are sparse. For example, the identification of key players involved in symbiosis establishment is still in its infancy: previous comparative transcriptomics in coral larvae were unable to find many candidates. One reason may be presumably low symbiont-to-host-cell ratios that masked signals from symbiont-carrying endodermal tissue^17^,^18^,^19^. Furthermore, many corals spawn only once annually, severely limiting larvae access and optimization of experiments.

The small sea anemone *Aiptasia* is an emerging model for coral symbiosis^20^. Similar to corals, *Aiptasia* produces symbiont-free offspring that then establish symbiosis with various *Symbiodinium* strains but not others, suggesting that “symbiosis specificity” is a common, lineage-independent phenomenon among symbiotic cnidarians^21^,^22^. Importantly, defined clonal lines are available for *Aiptasia* and for *Symbiodinium*, including axenic strains representing four of the nine described major *Symbiodinium* clades A-I^2^,^21^,^23^.

We recently established a robust protocol to induce *Aiptasia* spawning in the laboratory^23^. We then described when and where during larval development symbionts are phagocytosed by endodermal cells, defining reproducible experimental conditions for analyzing symbiont acquisition, and developed a set of experimental tools^24^. These resources, in conjunction with various transcriptomic, proteomic, and genomic resources for *Aiptasia*, *Symbiodinium*, and corals (for comparative analyses)^25^,^26^,^27^,^28^,^29^,^30^,^31^,^32^, provide the foundation to use *Aiptasia* larvae as a platform to study the molecular mechanisms of symbiosis establishment in cnidarians.

Here we further develop the *Aiptasia* larval system as a model for uncovering fundamental aspects of cnidarian symbiont selection at the molecular level. We directly compared symbiont selection patterns between larvae of *Aiptasia* and of two major reef-building coral species: *Acropora digitifera* and *Acropora tenuis.* We found that the overall patterns of “symbiosis specificity” are maintained between lineages, suggesting common underlying mechanisms of symbiont selection in *Aiptasia* and corals. Using *Aiptasia* larvae as a model, we find that both uptake into the gastric cavity and phagocytosis into the endoderm play a role in distinguishing between compatible and incompatible symbionts, and we identified a set of genes likely involved in symbiosis establishment by RNA-Seq. We conclude that *Aiptasia* is a powerful system to dissect the complex molecular mechanisms underlying initial symbiont selection.

## Results

### Symbiosis patterns are similar in *Aiptasia* and *Acropora*

**Figure 1:**
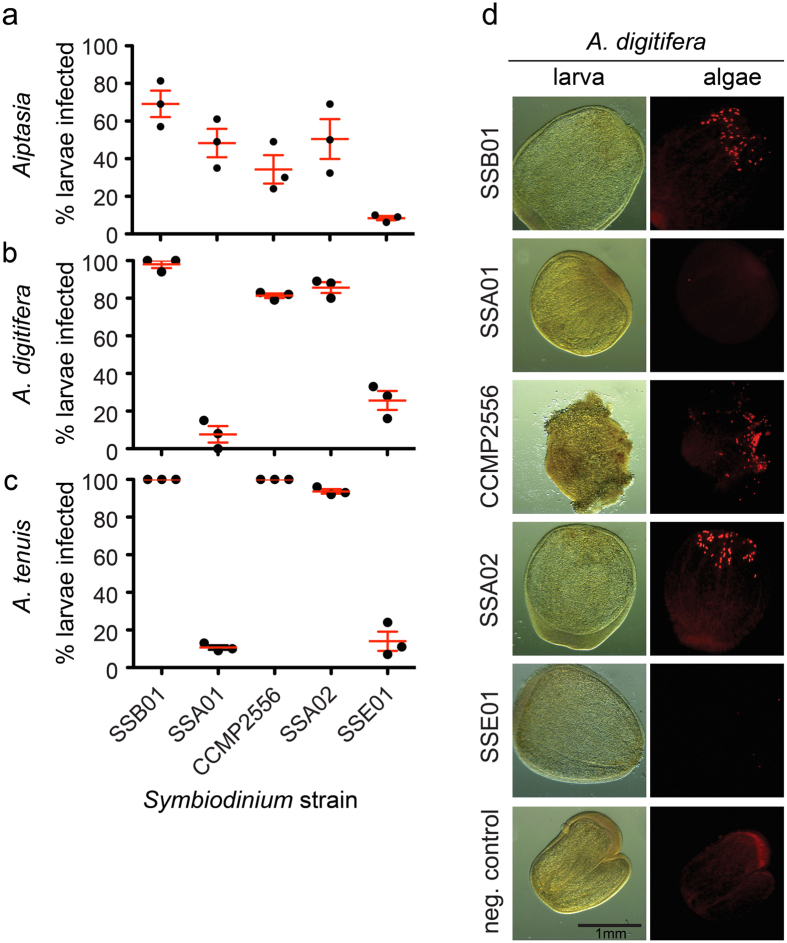
Symbiosis patterns are similar between *Aiptasia* and *Acropora*. (**a**–**c**) Percentage of infected larvae of *Aiptasia* (**a**) and the corals *Acropora digitifera* (**b**) and *Acropora tenuis* (**c**) after exposure to the indicated *Symbiodinium* strains at 10,000 algal cells/ml for 10 days. Strains used in this study are SSB01 (clade B), SSA01 (clade A), CCMP2556 (clade D), SSA02 (clade A), and SSE01 (clade E). Infected larvae contain one or more algal cells. Error bars are SEM of 3 replicate experiments. (**d**) Representative images of *Symbiodinium* infections in *A. digitifera* larvae. Left panels are brightfield images, right panels are red autofluorescence of algal photosynthetic pigments. Note the diffuse weak autofluorescence of larvae that is distinct from the bright puncta of the algae.

To date, a direct comparison of symbiosis establishment patterns between *Aiptasia* and corals is missing. To assess similarities and differences between *Aiptasia* and two major reef-building corals of the genus *Acropora*, *A. tenuis* and *A. digitifera*, we compared symbiont selection at early life stages using defined *Symbiodinium* strains. We incubated *Aiptasia* and coral larvae with four clonal, axenic *Symbiodinium* strains – SSA01, SSA02 (both clade A), SSB01 (clade B), SSE01 (clade E)^21^,^23^ – and the non-clonal, non-axenic strain CCMP2556 (clade D)^33^,^34^ for 10 days. We found that the strains SSA02, SSB01, and CCMP2556 efficiently infected hosts, whereas strain SSE01 was found in lower proportions of the larval populations (Fig. 1a–c). Strain SSA01^23^,^24^,^25^ was able to efficiently establish symbiosis with *Aiptasia* larvae but was rarely found in *Acropora* larvae (Fig. 1b–d). In line with the observation that the *Symbiodinium* strains SSA02, SSB01, and CCMP2556 efficiently infect coral larvae, microscopic analysis shows that many *Symbiodinium* cells were taken up per larva, especially when compared to strains SSE01 and SSA01 (Fig. 1d; Supplementary Fig. S1).

**Figure 2:**
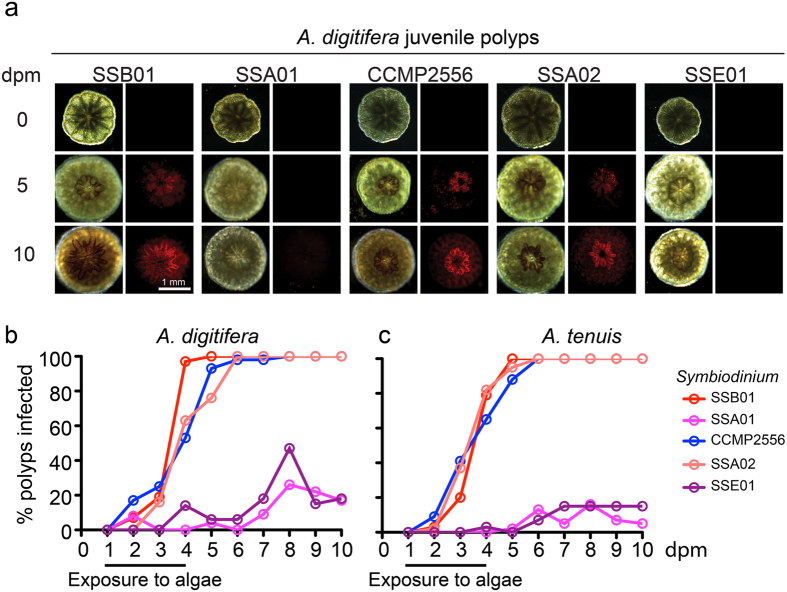
Symbiosis patterns are maintained between *Acropora* larvae and juvenile polyps. (**a**) Representative images of *Symbiodinium* infections in *A. digitifera* polyps. Left panels are brightfield images, right panels are red autofluorescence of algal photosynthetic pigments. dpm = days post-metamorphosis. (**b**,**c**) Percentage of infected juvenile polyps of *A. digitifera* (**b**) and *A. tenuis* (**c**) after exposure to the indicated *Symbiodinium* strains at 10,000 algal cells/ml for 4 days. Infected polyps contained visible algal cells.

Symbiosis establishment patterns are maintained between larva and polyp stages in *Aiptasia*^22^; to test whether such similarities hold true for *Acropora* larvae and juvenile polyps, we exposed juvenile *Acropora* polyps to the five different *Symbiodinium* strains for four days, after which algae were removed from the environment; progression of infection was monitored for six additional days (Fig. 2; Supplementary Fig. S2). We found that, as in conspecific larvae, polyps were efficiently infected by the *Symbiodinium* strains SSB01, SSA02, and CCMP2556 within the monitored time period (Fig. 2a). Symbiosis establishment occurred rapidly, with most polyps hosting algae after only 5 days and comparably robust populations after 10 days (Fig. 2a). Residential algal populations increased after the removal of environmental algae, indicating *in hospite* symbiont proliferation (Fig. 2a). *Symbiodinium* strains SSA01 and SSE01 failed to effectively infect polyps of either coral species, as these algae were nearly undetectable in polyps (Fig. 2; Supplementary Fig. S2).

**Figure 3:**
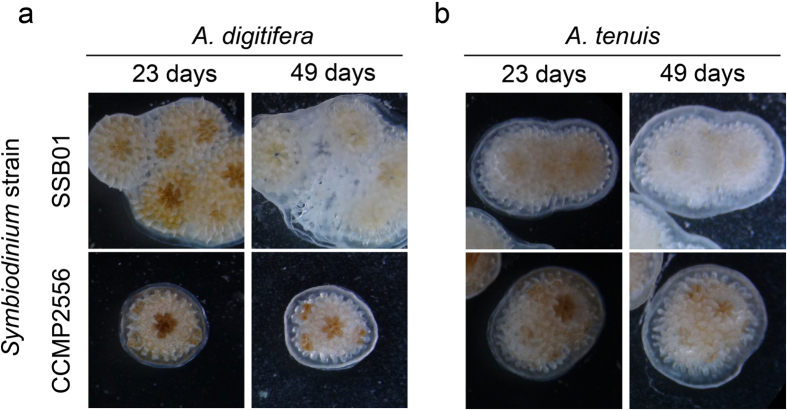
Long-term symbiosis patterns in juvenile *Acropora* polyps. Representative brightfield images of changes in residential *Symbiodinium* populations (brown coloration) over time in juvenile polyps of *A. digitifera* (**a**) and *A. tenuis* polyps (**b**) monitored for 49 days under laboratory conditions.

Symbiosis patterns established during early life stages may change over longer time periods^4^,^8^. Therefore, we tested whether the two symbiont types that infect *Acropora* most effectively, SSB01 and CCMP2556, were maintained over longer time periods in polyps under laboratory conditions. We exposed *A. tenuis* and *A. digitifera* larvae to either *Symbiodinium* strain for nine days, induced metamorphosis, and monitored the *in hospite* algal populations in the polyps with light microscopy 2, 23, and 49 days post-metamorphosis (dpm). At 2 dpm, the majority of polyps hosted symbionts: for *A. tenuis*, 35 of 35 polyps had SSB01 and 34 of 34 had CCMP2556; for *A. digitifera*, 12 of 16 had SSB01 and 26 of 27 had CCMP2556. At 23 dpm, 100% of the polyps were infected. At 49 dpm, 100% of polyps remained infected, although polyp budding and mortality led to different polyp numbers: for *A. tenuis*, 43 polyps with SSB01 and 41 with CCMP2556; for *A. digitifera*, 25 polyps with SSB01 and 18 with CCMP2556. Although all polyps were infected in the duration of the experiment, the populations of both CCMP2556 and SSB01 appeared to decrease during the monitored time period, SSB01 apparently more drastically than CCMP2556 (Fig. 3).

### Early acquisition steps are more efficient for compatible symbionts than incompatible symbionts

**Figure 4:**
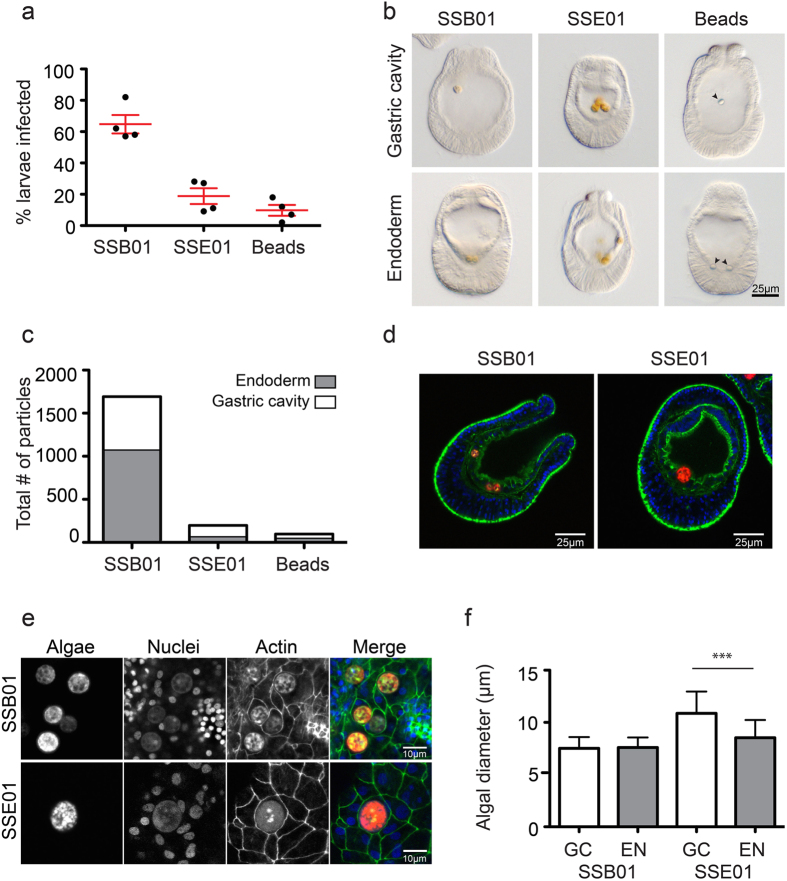
Early acquisition steps are more efficient for compatible symbionts than incompatible symbionts. (**a**) Percentage of *Aiptasia* larvae infected after exposure to the *Symbiodinium* strains SSB01 or SSE01 or inert polystyrene beads at 100,000 particles/ml for 4 or 5 days. Error bars are SEM of 4 replicate experiments. (**b**) Representative brightfield images of larvae from experiments in (**a**) used to distinguish between algae/beads in the gastric cavity or in the endoderm. Algae appear golden brown; arrows show beads. (**c**) Quantification of intra-larval localization of algae/beads in larvae from (**a**,**b**). (**d**) Representative confocal images of SSB01 and SSE01 algae integrated into the endodermal tissue. Colors are: autofluorescence of algal photosynthetic pigment (red), Hoechst-stained nuclei (blue), phalloidin-stained F-actin (green). (**e**) Representative confocal images of intracellular SSB01 and SSE01 algae. Colors of the merge are: autofluorescence of algal photosynthetic pigment (red), Hoechst-stained nuclei (blue), phalloidin-stained F-actin (green). (**f**) Quantification of cell sizes of SSB01 and SSE01 in the gastric cavity (GC) or in the endoderm (EN). Error bars are SEM, ***p < 0.0001 as determined by Student’s two-tailed unpaired *t*-test.

Our comparative analysis showed striking similarities in symbiont selection between *A. tenuis, A. digitifera*, and *Aiptasia*, with high compatibility of SSB01 and low compatibility of SSE01. To better understand the common principles underlying the broad preference for SSB01 over SSE01, we used *Aiptasia* larvae as a model and compared two distinct steps during the early phase of symbiosis establishment: uptake of symbionts into the gastric cavity and phagocytosis of symbionts into the endodermal tissue. We infected *Aiptasia* larvae 6–7 days post-fertilization (dpf) for four days with either SSB01, SSE01, or inert fluorescent polystyrene beads (7 μm diameter) and again found that SSB01 was taken up more efficiently by *Aiptasia* larvae (an average 65% of larvae contained one or more symbionts) than SSE01 and inert beads (19% and 10%, respectively) (Fig. 4a). When distinguishing the localization of algae inside larvae (Fig. 4b), we found that the majority of SSB01 algae appeared to be integrated in the endoderm (63%; 1072/1693), compared to only 33% (65/198) of SSE01 algae (Fig. 4c). Interestingly, of the beads that were taken up by larvae, a higher proportion (49%; 48/98) than SSE01 were found in the endoderm (Fig. 4c). Because SSE01 has been thought to be free-living^21^,^33^,^35^, its endodermal localization was surprising. We therefore imaged larvae at the tissue and cellular level using confocal microscopy to demonstrate that, indeed, SSB01 and SSE01 are found intracellularly in the endodermal cells (Fig. 4d,e).

In other systems, phagocytosis efficiency is dependent on size and shape of the phagocytosed particle, with larger particles being phagocytosed less efficiently than smaller ones^36^. As noted previously, SSE01 cells are relatively large, especially compared to SSB01 cells^33^,^37^ (Fig. 4b). To ask whether the cell size of SSE01 may correlate with phagocytosis efficiency, we compared cell sizes of these algae found in the gastric cavity and in the endoderm. SSE01 cells in the endoderm were significantly smaller than algae in the gastric cavity (8.5 μm vs. 11 μm), whereas such a difference could not be detected for SSB01 cells (Fig. 4f). Together, our results indicate that the compatible symbiont strain SSB01 is more efficiently taken up into the gastric cavity and phagocytosed into the endoderm, with the latter process potentially influenced by algal cell size.

### Identification of candidate genes involved in symbiosis by RNA-Seq

Identification and functional characterization of key players involved in symbiosis establishment and selection during larval stages is crucial to understand these processes at the molecular level. We therefore used RNA-Seq to compare gene expression between symbiotic and non-symbiotic *Aiptasia* larvae. *Aiptasia* larvae (5 dpf) were either infected with SSB01 or kept non-symbiotic as a control for five days^29^. We identified 19,771 genes (of a total of 26,039 genomic gene models) expressed in at least one of the four replicates (two symbiotic and two non-symbiotic samples) with an FPKM value over 0 (Fragments Per Kilobase reference per Million mapped reads). Of these genes, the difference in expression levels were significant for 351 genes between the two states (False Discovery Rate ≤ 0.1). The majority of differentially expressed genes (n = 219) are down-regulated in the symbiotic state, resulting in an average log2 fold change of −2.3. However, the log2 fold changes ranged from −10.9 to 10.8. (Supplementary Table S1). To assess and illustrate clustering and variation between replicates, gene expression data was plotted as a multidimensional scaling plot (MDS plot) (Supplementary Fig. S3).

**Figure 5:**
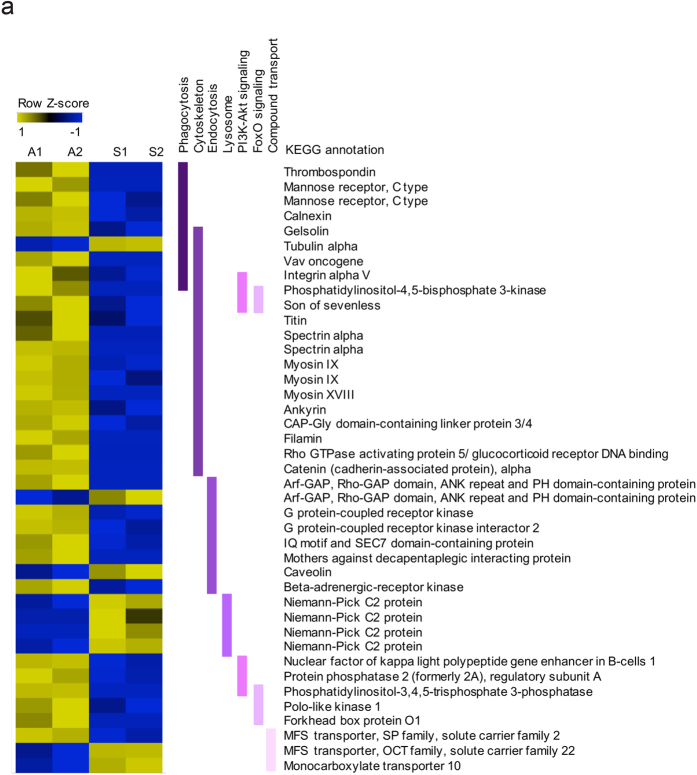
Differential expression of genes putatively involved in symbiosis establishment in *Aiptasia* larvae. Heatmap of 41 significantly differentially expressed genes. Gene expression was calculated over two replicates of aposymbiotic (A1, A2) and symbiotic (S1, S2) samples each. Up-regulation of gene expression is shown in yellow, down-regulation in blue. Genes were categorized by KEGG pathway annotations (purple bars) potentially involved in symbiosis establishment: Phagocytosis (ko04145: Phagosome, ko04666: Fc-gamma mediated phagocytosis), Cytoskeleton (ko04812: Eukaryotic cytoskeleton proteins, ko04810: Regulation of actin cytoskeleton), Endocytosis (ko04144), Lysosome (ko04142), PI3K-Akt signaling (ko04151), FoxO signaling (ko04068) and Compound transport. KEGG annotation was automated based on homology.

Recent transcriptomic and proteomic approaches in different systems have identified a set of symbiosis-specific candidate genes, and we found many similar genes among our list of differentially expressed genes (DEG); for example, the lysosomal Niemann-Pick disease type C2 (NPC2) protein^28^,^38^; transmembrane receptors that may play a role in symbiont recognition^29^,^39^,^40^; and small GTPases potentially involved in endocytotic vesicle transport during phagocytosis^41^,^42^,^43^ (Fig. 5). However, our dataset also identified many new genes encoding factors potentially involved in symbiosis establishment. Using the functional annotations of the Kyoto Encyclopedia of Genes and Genomes (KEGG)^44^, we found in total 41 DEGs related to phagocytosis, endocytosis, lysosomes, signaling pathways, cytoskeletal reorganization, and transport of compounds between partners. Among these genes, 9 genes are up- and 32 are down-regulated in the symbiotic state (Fig. 5). Thus, RNA-Seq of whole larvae is sufficiently sensitive to identify many genes putatively involved in symbiosis establishment.

## Discussion

“Symbiosis specificity”, defined as non-random host/symbiont combinations, may have broad ecological implications for coral reef ecosystems; however, this remains a matter of debate, with interpretations of the symbiosis ranging from specific and evolutionarily fixed to largely random and highly adaptive (e.g. during environmental change)^7^,^45^,^46^,^47^,^48^,^49^,^50^,^51^,^52^,^53^. Reaching a unified understanding of fundamental principles is difficult because of the complexity of the phenomenon: it is likely influenced by coral taxa, developmental stage, varying microhabitats of corals and symbionts, and local availability of symbionts. These factors, together with limited experiments under controlled laboratory conditions to standardize experimental design and replication, slow the progress of directly testing influences of host and symbiont. The molecular mechanisms underlying “symbiosis specificity” are likewise unclear and remain impossible to dissect without molecular tools and approaches.

To dissect “symbiosis specificity” using *Aiptasia* larvae as a model, we provide here the first direct comparison of symbiont uptake specificity in *Aiptasia* and *Acropora* under laboratory conditions using defined *Symbiodinium* strains. We tested two strains (SSB01 and SSA01) originating from *Aiptasia*^21^,^23^,^25^, two strains (SSA02 and CCMP2556) isolated from corals^21^, and the presumably free-living strain SSE01^21^,^33^,^35^. We find that patterns of symbiont selectivity are very similar between these taxa, indicating low lineage-constrained selectivity for *Symbiodinium* during early development stages (larval and juvenile polyp) independent of whether the algal strain originated from *Aiptasia* or a coral host. Of the *Symbiodinium* strains tested, three (SSB01, SSA02 and CCMP2556) are positively selected and one (SSE01) is negatively selected by *Aiptasia* and both Acroporids, even when the latter strain is present at high concentrations. This indicates that fundamental mechanisms of positive and negative symbiont selection are generally conserved between *Aiptasia* and *Acropora* during early life stages. SSA01 is a notable exception: it is efficiently acquired by progeny of its original host (*Aiptasia*) but rejected by both *Acropora* species tested. This comparative dataset is an important step towards dissecting fundamental and conserved principles (as well as differences) underlying host/symbiont compatibility and incompatibility in cnidarians.

Symbiosis establishment is a complex process comprising multiple steps, including symbiont uptake into the gastric cavity, phagocytosis by host cells, integration into host cell function, long-term persistence, and proliferation^15^. Each step, alone or in conjunction with others, may play a role during the establishment of suitable host/symbiont combinations. Further, each step may be influenced by specific molecular mechanisms as well as physical factors (36,54, this study). In the first step, encounter efficiency may be important for initial uptake into the gastric cavity; for example, we have previously shown that symbiont uptake efficiency is concentration-dependent in *Aiptasia* larvae^24^. However, symbionts are presumably sparse in the natural environment and direct chemical attraction may facilitate host/symbiont meeting^54^. Once inside the gastric cavity, algae and host cell-cell contact is important to trigger phagocytosis. To date, it is unclear whether symbiont phagocytosis is restricted to certain cells with distinct receptors or whether it is an unspecific, evolutionarily conserved feeding mechanism in nutritive cells^15^,^55^. Many cell types are capable of engulfing particles, but phagocytosis requires fundamental changes in cell shape and architecture and, accordingly, particle size and shape directly influence cells’ phagocytosis efficiencies^36^. Despite having entered host cells, symbionts may still fail to populate hosts; various examples indicate that coral larvae initially take up heterologous symbionts that are ultimately not maintained over longer time periods^12^,^13^ (Fig. 3). It is unknown whether this lack of long-term compatibility is due to symbiont expulsion, digestion, or a lack of proliferation capacities.

Indeed, our analyses in *Aiptasia* larvae directly comparing the broadly compatible symbiont strain SSB01 and the incompatible strain SSE01 confirm the collective influence of multiple steps for the efficiency of symbiont acquisition. Despite our observation that SSE01 cells are phagocytosed into *Aiptasia* larvae endodermal cells, we repeatedly find virtually no larvae stably infected with SSE01 under the tested conditions. This failure stands in contrast to the consistent high infection rates of SSB01 symbionts in larvae. SSB01 is taken up into the gastric cavity more efficiently than SSE01 (alternatively, the SSE01 expulsion rate may be higher); phagocytosis of SSB01 is more efficient than that of SSE01; and while the number of SSB01 algae inside larvae increases over time, SSE01 fail to persist in larvae at detectable levels (22; this study). Phagocytosis efficiency may be directly related to symbiont size, as SSE01 is phagocytosed at the lowest rates, yet our comparison of SSB01, SSE01, and small inert beads indicates that entering or persisting in the gastric cavity is not (Fig. 4a,c). It is noteworthy that SSE01, similar to other *Symbiodinium* types in Clade E, is thought to be free-living^21^,^33^,^35^. Our data suggest that the inability of SSE01 to establish stable symbiosis in cnidarians is not governed by the inhibition of phagocytosis into host cells, but may rather be a consequence of the inability of the two partners to initiate a functional molecular crosstalk after intracellularization. The direct involvement of more complex molecular mechanisms during symbiont selection is supported by our observation that SSA01 acceptance by cnidarian hosts is opposite for *Aiptasia* and Acroporids: *Aiptasia* larvae take up SSA01 cells with high efficiency, but *Acropora tenuis* and *Acropora digitifera* do not (Fig. 1).

In the future, it will be also interesting to extend the analysis of changes in “symbiosis specificity” over longer time periods. Other studies have shown that juvenile *A. tenuis* larvae exhibit different specificity throughout ontogeny, with nonhomolgous symbiont types (in clade D) being taken up first and replaced only after several months/years by the homologous symbionts (in clade C) that dominate adult colonies^5^,^34^,^56^. Our observations that populations of nonhomologous symbiont strains SSB01 (clade B) and CCMP2556 (clade D) declined over time in *Acroprora* polyps are consistent with this phenomenon (Fig. 3). However, *Acropora* polyps are notoriously difficult to keep in the laboratory, and the polyps we monitored under laboratory conditions suffered substantial mortality. Further, the low number of polyps in our experiment prevents us from drawing hard conclusions: additional experiments are needed under controlled and optimized growth conditions for juvenile *Acropora*. Alternatively, similar long-term experiments could be done with *Aiptasia* and defined *Symbiodinium* strains under controlled conditions, which would greatly increase reproducibility and comparability and further reveal the complex dynamics of symbiosis specificity over time. Likewise, *Aiptasia* may help reveal the influence of environmental factors (e.g. elevated temperature) on symbiosis specificity, further highlighting the need for a laboratory-based model system to dissect “symbiosis specificity” in a systematic, controlled way.

To dissect distinct molecular mechanisms involved in “symbiosis specificity” and symbiosis establishment in general, the identification of key players is crucial. Here we used RNA-Seq as a proof-of-concept to identify symbiosis-specific genes in *Aiptasia* larvae, and we found over 300 genes that are differentially expressed in symbiotic versus non-symbiotic larvae. Notably, a suite of those genes are involved in pathways and biological processes that were previously identified to play a role in the cnidarian-dinoflagellate endosymbiosis, validating this approach^28^,^29^,^32^,^39^,^40^,^41^,^42^,^43^. RNA-Seq is an easy, rapid, and cost-effective technique, and in the future it can be combined with cell biological analyses to reveal the functions of identified candidates in the symbiosis.

We propose that *Aiptasia* larvae, together with the suite of compatible and incompatible symbiont strains, constitute a powerful platform to elucidate fundamental mechanisms of the distinct steps involved in symbiosis establishment in cnidarians. We envision exploiting the *Aiptasia* larvae model by taking advantage of the easily controllable laboratory system and available molecular and cell biological tools, including RNA-Seq, to mechanistically dissect how stable host/symbiont combinations are established. Moreover, we can begin to dissect the various contributions of environmental conditions, host and symbiont genotype, and ontogeny to the phenomenon of “symbiosis specificity”. Ultimately, such laboratory experiments may also help to better understand and predict the potential of certain symbiotic associations for the resilience and adaptive capacities of coral reefs to environmental change.

## Methods

### Collection and maintenance of *Acropora* planula larvae

Colonies of *Acropora digitifera* and *Acropora tenuis* were collected with permission by the Okinawa prefecture (#27-1) at Sesoko Island (26°37’41”N, 127°51’38”E, Okinawa, Japan). Corals were kept in tanks with running natural seawater and under partially shaded natural light at Sesoko Marine Station (University of Ryukyus, Okinawa, Japan). After spawning on May 31 2015, bundles of symbiont-free gametes from multiple colonies of each coral species were mixed and the resulting planula larvae were maintained in plastic bowls at approximately 1000 larvae/L in 10 μm-filtered natural seawater (FNSW). FNSW was exchanged daily.

### *Aiptasia* culture conditions and spawning induction

Spawning of *Aiptasia* clonal lines CC7 and F003 (for symbiosis establishment studies) and clonal lines CC7 and H2 (for transcriptomic comparisons) was induced as previously described^23^. *Aiptasia* larvae were kept in filter-sterilized artificial seawater (FASW) in glass beakers at 26 °C on a 12 h light:12 h dark (12L:12D) cycle.

### *Symbiodinium* cultures and conditions

For infection of cnidarian hosts, we used the following clonal and axenic *Symbiodinium* strains: SSB01 (clade B), SSA01 (clade A), SSA02 (clade A), and SSE01 (clade E)^21^,^23^ as well as the non-clonal, non-axenic *Symbiodinium* culture CCMP2556 (clade D) from the scleractinian coral *Orbicella faveolata* (formerly *Montastrea faveolata*)^33^ purchased from the National Center for Marine Algae and Microbiota (NCMA, Bigelow Laboratory for Ocean Sciences, Maine, USA). All cultures were grown in cell culture flasks in IMK medium^57^ at 26 °C on a 12L:12D cycle under 20–25 μmol m^−2^ s^−1^ of photosynthetically active radiation (PAR), as measured with an Apogee PAR quantum meter (MQ-200; Apogee, Logan, USA).

### Symbiosis establishment experiments in *Acropora* and *Aiptasia*

#### Aiptasia larvae

For infection experiments, *Aiptasia* larvae 2 days post-fertilization (dpf) were distributed into 6-well plates at 300 larvae in 5 ml of FASW per well. *Symbiodinium* cells were added to each well at a final concentration of 10,000 algal cells/ml; FASW was used as a negative control. Three biological replicates (e.g. spawning crosses) were used per algae/host combination. Plates were kept in incubators at 26 °C under white fluorescent bulbs at 20–25 μmol m^−2^ s^−1^ on a 12L:12D cycle with regular exchange of FASW; *Symbiodinium* cells were re-added after each wash. After ten days of exposure to *Symbiodinium*, larvae were fixed for 30 min in 4% formaldehyde in seawater, washed 3 times in PBS, and mounted in 87% glycerol in PBS for analysis. Over 45 *Aiptasia* larvae were counted per algae/host replicate.

The experiments in Fig. 4 were carried out with the following changes: larvae were 6–7 dpf at start of infection; *Symbiodinium* cells of strains SSB01 and SSE01 and inert polysterene fluorescent beads (#C36950, Thermo Fisher Scientific) were added at a final concentration of 100,000 particles/ml; larvae were fixed for analysis after 4–5 days of exposure to algal cells. Over 85 *Aiptasia* larvae were counted per algae/host replicate.

#### Acropora larvae

At 4 dpf, *Acropora* larvae of either coral species were distributed into 24-well cell culture plates, with 30 larvae in 2 ml FNSW per well. *Symbiodinium* cells were added to each well at a final concentration of 10,000 algal cells/ml; FNSW was used as a negative control. Three technical replicates (i.e. wells) were used per algae/host combination. Plates were kept at ∼25 °C under ambient room light (∼9–12 μmol m^−2^ s^−1^) on an approximate 12L:12D cycle. Larvae were washed with FNSW every one or two days as appropriate; *Symbiodinium* cells were re-added after each wash. After ten days of exposure to *Symbiodinium*, larvae were mounted in FNSW on glass slides with glass coverslips for analysis. An average of 22 larvae were counted per technical triplicate; for details, see Supplementary Table S2.

#### *Acropora* polyps: short-term exposure

At 6 dpf, larvae of either species were induced to metamorphose and settle in 6-well plates through overnight incubation in 5 ml FNSW supplemented with 1 μM Hym-248 neuropeptide^58^. The following day, 30–60 polyps had metamorphosed and settled per plate (∼5–10 polyps in per well). *Symbiodinium* cells were added to each well at a final concentration of 10,000 algal cells/ml; FNSW was used as a negative control. Plates were kept at ∼25 °C under ambient light (∼9–12 μmol m^−2^ s^−1^) on an approximate 12L:12D cycle with daily exchanges of FNSW; *Symbiodinium* cells were re-added after each wash. *Symbiodinium* exposure proceeded for four days, after which environmental algae were removed by exchanging FNSW without re-addition of *Symbiodinium*. Polyps were monitored daily for 10 days total by microscopy. An average of 41 polyps were counted per host/algae combination; for details, see Supplementary Table S3.

#### *Acropora* polyps: long-term exposure

At 5 dpf, larvae of either species were distributed into plastic bowls at 250 larvae per 200 ml FNSW. *Symbiodinium* cells of strains SSB01 or CCMP2556 was added to each bowl at a final concentration of 10,000 algae cells/ml; FNSW was used as a negative control. Bowls were kept on the bench at ∼25 °C under ambient light (∼9–12 μmol m^−2^ s^−1^) on an approximate 12L:12D cycle. At 12 dpf larvae were package at a density of ~1000 larvae/L in 50 ml conical tubes and overnight shipped to the Hatta lab, Tokyo. At 14 dpf, environmental algae were removed by exchanging FNSW without re-addition of algae and metamorphosis was induced as described above. Polyps that had successfully metamorphosed and settled by the following day were included in subsequent monitoring: for *A. tenuis*, 35 polyps exposed to SSB01 and 34 polyps exposed to CCMP2556; for *A. digitifera*, 15 polyps exposed to SSB01 and 27 polyps exposed to CCMP2556. The polyps were then washed with daily exchange of seawater, which was a 50%/50% mixture of FNSW sterilized at 105 °C for 3 min and artificial seawater (Coral Pro, RedSea). Polyps were qualitatively assessed as hosting symbionts by light microscopy and photography at 2, 23, and 49 days post-metamorphosis (dpm). At 23 dpm, polyp numbers were those from the initial metamorphosis; at 49 dpm, budding and mortality led to new population sizes: for *A. tenuis*, 43 polyps with SSB01 and 41 with CCMP2556; for *A. digitifera*, 25 polyps with SSB01 and 18 with CCMP2556.

### Microscopy

*Acropora* larvae and polyps were analyzed using a Leica S8APO stereoscope equipped with a Leica MC170 HD color camera. Endogenous autofluorescence of *Symbiodinium* photosynthetic pigments was visualized using the Leica S8APO stereoscope in combination with a Stereomicroscope Fluorescence Adapter kit (#SFA-LFS-Green, NightSea, USA). Microscopic analysis of *Aiptasia* larvae was carried out with a Nikon Eclipse Ti inverted microscope using Differential Interference Contrast (DIC) and a Nikon Plan Fluor 20x dry lens. Microscopic images of *Aiptasia* larvae were captured with a Nikon Eclipse 80i microscope using DIC and a Digital Sight DS-U1 color camera (Nikon Instruments).

For confocal imaging, *Aiptasia* larvae 4 dpf were infected for 4 days and then fixed in 4% formaldehyde in FASW for 30 min, followed by three washes in PBS containing 0.2% Triton X-100 (PBT). Approximately 50–100 larvae were transferred to 0.2 ml reaction tubes and permeabilized in 0.01 mg/ml Proteinase K (#03115879001, Roche Diagnostics) in 1x PBS for 8 min. Permeabilization was stopped by washing larvae in 2 mg/ml glycine in PBT twice for 15 min each. Larvae were post-fixed in 4% formaldehyde in PBT for 20 min and then washed twice in PBT and twice in PBS, 15 min per wash. Larvae were incubated in Phalloidin-Atto 565 (#94072, Sigma-Aldrich) diluted 1:200 in PBS for 60 min on a rotor (Intelli mixer #7-0045, NeoLab) at 20 rpm. Larvae were washed twice in PBT before incubation in 10 μg/ml Hoechst in buffer (Tris-buffered saline, pH 7.4; 0.1% Triton X-100; 2% bovine serum albumin; 0.1% sodium azide) for 15 min. After three washes in PBT for 15 min each, larvae were mounted in 87% glycerol in PBS containing 2.5 mg/ml DABCO (1,4-Diazabicyclo[2.2.2]octan, #D27802, Sigma-Aldrich). Confocal images were acquired using a Nikon A1R confocal microscope with a Nikon Plan Apo 60x oil immersion objective (NA = 1.4) and Nikon Elements Software. Image processing and maximum projections of Z-stacks were performed using Fiji^59^.

### Analysis of differential gene expression

#### RNA isolation and sequencing

For the analysis of differentially expressed genes, the *Aiptasia* larval RNA-Seq datasets described in Baumgarten *et al.*^29^ were obtained from the NCBI SRA database, with two replicates for aposymbiotic (accession no.: SRX757531) and symbiotic (accession no.: SRX757532) larvae each. Briefly, larvae 3–4 dpf were infected with *Symbiodinium* strain SSB01 at a concentration of 2.5–5 × 10^4^ algae/ml for 5–6 days (FASW as negative control). Two separate crosses were used for duplicate pairs, with cross 1 containing 6,500 larvae per treatment and cross 2 containing 8,400 larvae per treatment. For all samples, total RNA was extracted using TriZol (#15596, Thermo Fisher Scientific) and the mRNA was purified using Dynabeads oligo(dT)25 (#61002, Thermo Fisher Scientific). The sequencing libraries were prepared with the NEBNext Ultra Directional RNA Library Prep Kit (#E7420, NEB) with 180 bp insert sizes and sequenced on an Illumina HiSeq2000 at 2 × 101 bp read length. As a mapping reference for the calculation of transcript abundances, we used the *Aiptasia* genomic gene set (NCBI Genome ID: 40858, Accession: LJWW01000000).

Sequencing adaptors and low quality reads were trimmed and filtered from the sequence reads using Trimmomatic^60^. The reads were mapped to the reference genomic gene set using bowtie2^61^ with settings “-a -t -X 500–no-unal–rdg 6,5–rfg 6,5–score-min L,-.6,-.4–no-discordant–no-mixed–phred33”. Read count abundances were calculated using eXpress^62^ with settings “–rf-stranded” and a read count table was generated using a custom perl script.

#### Statistical analysis

Significantly differentially expressed genes (DEGs) were subsequently calculated in R^63^ using the package edgeR^64^. Specifically, to test for differential expression of genes between symbiotic and aposymbiotic *Aiptasia* larvae, first the variation of gene expression between replicates was determined. Two types of variation contribute to the total variation in RNA-Seq experiments and are expressed as follows: 1) technical coefficient of variation, which describes the measurement error derived from the uncertainty with which the abundance of every gene is estimated from the sequencing platform. This variation decreases with increasing total counts for each gene in an RNA sample and can be distinguished from 2) the biological coefficient of variation (BCV). The latter denotes the variation of the true, unknown expression of each gene among biological replicates, which remains even at indefinite sequencing depth^65^. This represents the most important source of variation in RNA-Seq experiments and is calculated from the biological replication prior to the test of differential expression between treatments^66^. Although higher confidence estimates of BCV might be obtained with higher numbers of biological replicates, both the common dispersion (i.e. BCV^2^) as well as the gene-specific dispersion could be calculated robustly among the four biological replicates (i.e. n = 2 for both aposymbiotic and symbiotic larvae) using the functions “estimateCommonDisp()” and “estimateTagwiseDisp()” using the package edgeR, respectively. Differential expression was subsequently calculated using the function “exactTest()” within the edgeR package and a multidimensional scaling (MDS) plot of differentially expressed genes (FDR ≤ = 0.1) was plotted using the function “plotMDS()”. The distances between pairs of RNA samples in the MDS plot correspond to the leading log2 fold changes, which is the average (root mean square) of the largest absolute log2 fold changes^64^.

#### Gene annotation

KEGG pathway identifiers of the differentially expressed genes were obtained from the gene annotations. To sort DEGs into higher level biological processes, we used the automated KEGG pathway annotations for *Aiptasia*^29^. DEGs were sorted by their KEGG annotations into the processes ‘Phagocytosis’ (ko04145: Phagosome, ko04666: Fc-gamma mediated phagocytosis), ‘Cytoskeleton’ (ko04812: Eukaryotic cytoskeleton proteins, ko04810: Regulation of actin cytoskeleton), ‘Endocytosis’ (ko04144), ‘Lysosome’ (ko04142), ‘PI3K-Akt signaling’ (ko04151), ‘FoxO’ (ko04068) as well as ‘Compound transport’. The log2 fold changes of DEGs belonging to either of these groups were calculated with the predFC function in edgeR, normalized to Z-scores following the formula z = (x-u)/s (z = Z score; x = fold change; u = mean fold change across all replicates; s = standard deviation across all replicates) and visualized as a heatmap in MeV^67^.

## Additional Information

**How to cite this article**: Wolfowicz, I. *et al.* *Aiptasia* sp. larvae as a model to reveal mechanisms of symbiont selection in cnidarians. *Sci. Rep.* **6**, 32366; doi: 10.1038/srep32366 (2016).

## Supplementary Material

Supplementary Information

Supplementary Dataset S1
