# Supplementary material for: Aiptasia sp. larvae as a model to reveal mechanisms of symbiont selection in cnidarians
Source: Sci Rep. 2016 Sep 1;6:32366. doi: 10.1038/srep32366 (PMC5007887; doi:10.1038/srep32366)
Supplement: Supplementary Information [file srep32366-s1.pdf]

SUPPLEMENTARY INFORMATION

Aiptasia sp. larvae as a model to reveal mechanisms of symbiont selection in cnidarians

Authors: I. Wolfowicz, S. Baumgarten, P.A. Voss, E.A. Hambleton, C.R. Voolstra, M. Hatta, A. Guse

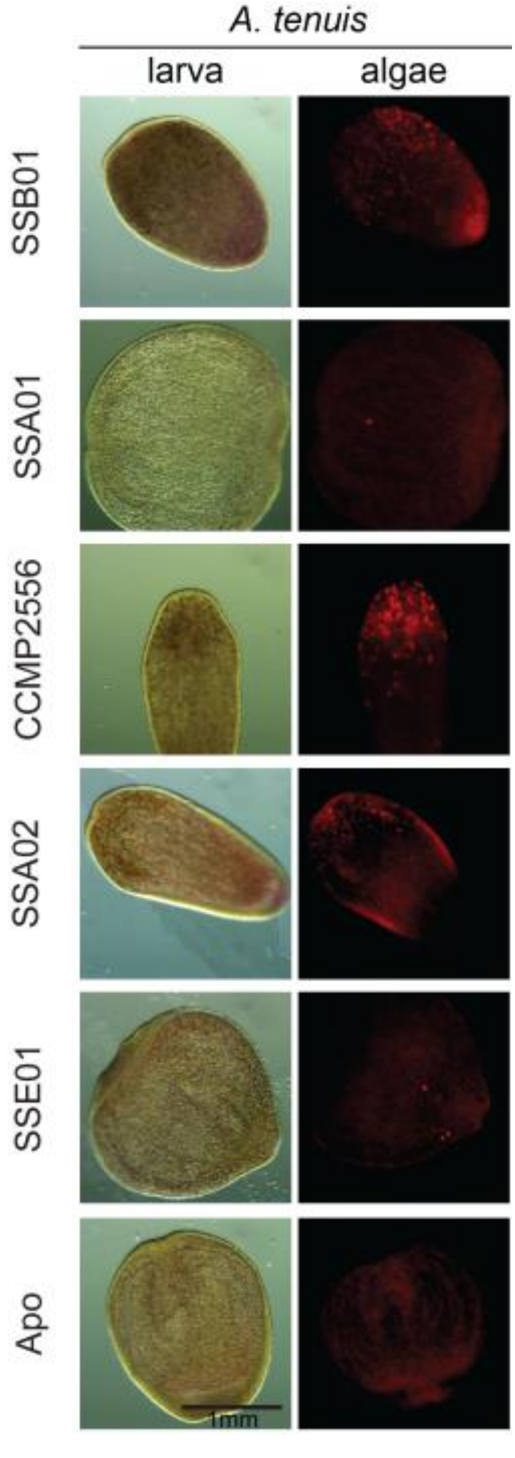

Supplementary Figure S1: Representative images of *Symbiodinium* infections in *A. tenuis* larvae with *Symbiodinium* strains SSB01, SSA01, CCMP2556, SSA02, SSE01 and the aposymbiotic control. Left panels are brightfield images, right panels are red autofluorescence of algal photosynthetic pigments. Note the diffuse weak autofluorescence of larvae that is distinct from the bright puncta of the algae.

SUPPLEMENTARY INFORMATION
Aiptasia sp. larvae as a model to reveal mechanisms of symbiont selection in cnidarians
Authors: I. Wolfowicz, S. Baumgarten, P.A. Voss, E.A. Hambleton, C.R. Voolstra, M. Hatta, A. Guse

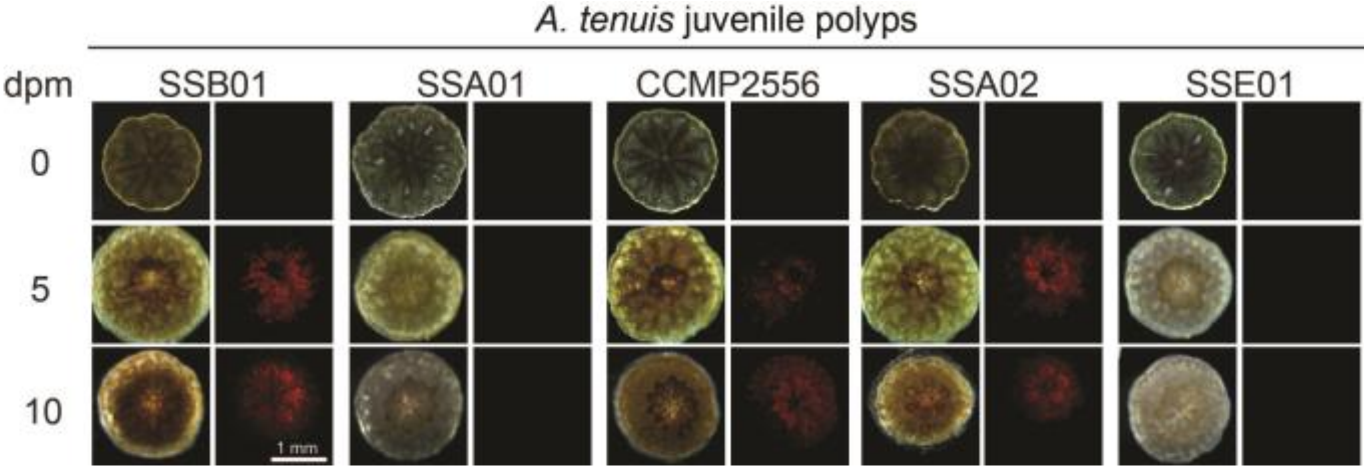

Supplementary Figure S2: Representative images of *Symbiodinium* infections in *A. tenuis* polyps. Left panels are brightfield images, right panels are red autofluorescence of algal photosynthetic pigments. dpm = days post-metamorphosis.

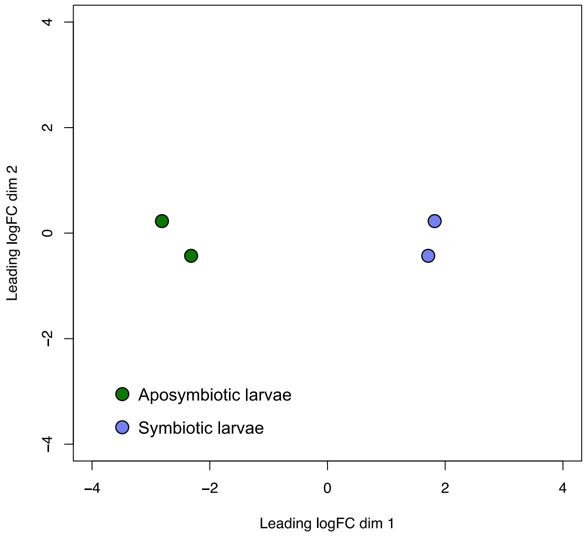

Supplementary Figure S3: Multidimensional scaling plot showing the replicate clustering along the primary and secondary leading log2 fold change (LFC) axes of differentially expressed genes ($n= 351$, $FDR \leq 0.1$).

SUPPLEMENTARY INFORMATION
Aiptasia sp. larvae as a model to reveal mechanisms of symbiont selection in cnidarians
Authors: I. Wolfowicz, S. Baumgarten, P.A. Voss, E.A. Hambleton, C.R. Voolstra, M. Hatta, A. Guse

Supplementary Table S2: Quantification of *Acropora* larvae infections with SSB01, SSA01, SSA02, SSE01, CCMP2556 and the aposymbiotic control.

A. tenuis	Triplicate #	# Larvae	# Larvae infected	% Larvae infected
SSB01	1	26	26	100
	2	25	25	100
	3	24	24	100

A. digitifera	Triplicate #	# Larvae	# Larvae infected	% Larvae infected
SSB01	1	13	13	100
	2	16	16	100
	3	17	16	94

A. tenuis	Triplicate #	# Larvae	# Larvae infected	% Larvae infected
SSA01	1	23	3	13
	2	30	3	10
	3	22	2	9

A. digitifera	Triplicate #	# Larvae	# Larvae infected	% Larvae infected
SSA01	1	20	3	15
	2	12	1	8
	3	14	0	0

A. tenuis	Triplicate #	# Larvae	# Larvae infected	% Larvae infected
SSA02	1	27	25	93
	2	26	24	92
	3	24	23	96

A. digitifera	Triplicate #	# Larvae	# Larvae infected	% Larvae infected
SSA02	1	16	14	88
	2	18	16	89
	3	10	8	80

A. tenuis	Triplicate #	# Larvae	# Larvae infected	% Larvae infected
SSE01	1	28	3	11
	2	28	2	7
	3	21	5	24

A. digitifera	Triplicate #	# Larvae	# Larvae infected	% Larvae infected
SSE01	1	18	6	33
	2	19	3	16
	3	18	5	28

A. tenuis	Triplicate #	# Larvae	# Larvae infected	% Larvae infected
CCMP 2556	1	29	29	100
	2	18	18	100
	3	31	31	100

A. digitifera	Triplicate #	# Larvae	# Larvae infected	% Larvae infected
CCMP 2556	1	29	24	83
	2	28	23	82
	3	28	22	79

A. tenuis	Triplicate #	# Larvae	# Larvae infected	% Larvae infected
Apo	1	27	0	0
	2	22	0	0
	3	22	0	0

A. digitifera	Triplicate #	# Larvae	# Larvae infected	% Larvae infected
Apo	1	18	0	0
	2	22	0	0
	3	25	0	0

SUPPLEMENTARY INFORMATION
Aiptasia sp. larvae as a model to reveal mechanisms of symbiont selection in cnidarians
Authors: I. Wolfowicz, S. Baumgarten, P.A. Voss, E.A. Hambleton, C.R. Voolstra, M. Hatta, A. Guse

Supplementary Table S3: Quantification of *Acropora* polyps infections with SSB01, SSA01, SSA02, SSE01, CCMP2556 and the aposymbiotic control. dpm = days post-metamorphosis.

A. tenuis	dpm	# polyps	# polyps infected	% polyps infected
SSB01	1	66	0	0
	2	66	2	3
	3	60	12	20
	4	58	46	79
	5	58	58	100
	6	57	57	100
	7	57	57	100
	8	53	53	100
	9	53	53	100
	10	53	53	100

A. digitifera	dpm	# polyps	# polyps infected	% polyps infected
SSB01	1	44	0	0
	2	44	3	7
	3	43	8	19
	4	33	32	97
	5	33	33	100
	6	32	32	100
	7	32	32	100
	8	32	32	100
	9	32	32	100
	10	32	32	100

A. tenuis	dpm	# polyps	# polyps infected	% polyps infected
SSA01	1	56	0	0
	2	56	0	0
	3	56	0	0
	4	56	0	0
	5	56	1	2
	6	56	7	13
	7	55	3	5
	8	55	9	16
	9	55	4	7
	10	55	3	5

A. digitifera	dpm	# polyps	# polyps infected	% polyps infected
SSA01	1	36	0	0
	2	36	3	8
	3	30	0	0
	4	26	0	0
	5	23	1	4
	6	23	0	0
	7	23	2	9
	8	23	6	26
	9	23	5	22
	10	23	4	17

A. tenuis	dpm	# polyps	# polyps infected	% polyps infected
SSA02	1	59	0	0
	2	59	1	2
	3	57	21	37
	4	56	46	82
	5	56	53	95
	6	56	56	100
	7	55	55	100
	8	55	55	100
	9	55	55	100
	10	55	55	100

A. digitifera	dpm	# polyps	# polyps infected	% polyps infected
SSA02	1	35	0	0
	2	35	0	0
	3	31	5	16
	4	30	19	63
	5	29	22	76
	6	14	14	100
	7	11	11	100
	8	9	9	100
	9	9	9	100
	10	9	9	100

SUPPLEMENTARY INFORMATION
Aiptasia sp. larvae as a model to reveal mechanisms of symbiont selection in cnidarians
Authors: I. Wolfowicz, S. Baumgarten, P.A. Voss, E.A. Hambleton, C.R. Voolstra, M. Hatta, A. Guse

Supplementary Table S3 (continued): Quantification of *Acropora* polyps infections with SSB01, SSA01, SSA02, SSE01, CCMP2556 and the aposymbiotic control. dpm = days post-metamorphosis.

A. tenuis	dpm	# polyps	# polyps infected	% polyps infected
SSE01	1	41	0	0
	2	41	0	0
	3	32	0	0
	4	32	1	3
	5	27	0	0
	6	27	2	7
	7	27	4	15
	8	27	4	15
	9	27	4	15
	10	27	4	15

A. digitifera	dpm	# polyps	# polyps infected	% polyps infected
SSE01	1	37	0	0
	2	37	0	0
	3	35	0	0
	4	35	5	14
	5	35	2	6
	6	35	2	6
	7	34	6	18
	8	34	16	47
	9	34	5	15
	10	34	6	18

A. tenuis	dpm	# polyps	# polyps infected	% polyps infected
CCMP 2556	1	54	0	0
	2	54	5	9
	3	51	21	41
	4	48	31	65
	5	48	42	88
	6	48	48	100
	7	48	48	100
	8	48	48	100
	9	48	48	100
	10	48	48	100

A. digitifera	dpm	# polyps	# polyps infected	% polyps infected
CCMP 2556	1	42	0	0
	2	42	7	17
	3	40	10	25
	4	40	21	53
	5	40	37	93
	6	40	39	98
	7	40	39	98
	8	40	40	100
	9	40	40	100
	10	40	40	100

A. tenuis	dpm	# polyps	# polyps infected	% polyps infected
Apo	1	50	0	0
	2	50	0	0
	3	49	0	0
	4	49	0	0
	5	48	0	0
	6	48	0	0
	7	44	0	0
	8	44	0	0
	9	41	0	0
	10	41	0	0

A. digitifera	dpm	# polyps	# polyps infected	% polyps infected
Apo	1	39	0	0
	2	39	0	0
	3	38	0	0
	4	38	0	0
	5	38	0	0
	6	38	0	0
	7	38	0	0
	8	38	0	0
	9	38	0	0
	10	38	0	0
